# Mechanism and comparison of needle-type non-thermal direct and indirect atmospheric pressure plasma jets on the degradation of dyes

**DOI:** 10.1038/srep34419

**Published:** 2016-10-06

**Authors:** Pankaj Attri, Maksudbek Yusupov, Ji Hoon Park, Lakshmi Prasanna Lingamdinne, Janardhan Reddy Koduru, Masaharu Shiratani, Eun Ha Choi, Annemie Bogaerts

**Affiliations:** 1 Plasma Bioscience Research Center/Department of Electrical and Biological Physics, Kwangwoon University, Seoul 139-701, Korea; 2 Graduate School of Information Science and Electrical Engineering, Kyushu University, Fukuoka, 819-0395, Japan; 3 Research Group PLASMANT, Department of Chemistry, University of Antwerp, Universiteitsplein 1, B-2610 Antwerp, Belgium; 4 Department of Environmental Engineering, Kwangwoon University, Seoul, 139-701, Republic of Korea; 5 Graduate School of Environmental Studies, Kwangwoon University, Seoul, 139-701, Republic of Korea

## Abstract

Purified water supply for human use, agriculture and industry is the major global priority nowadays. The advanced oxidation process based on atmospheric pressure non-thermal plasma (NTP) has been used for purification of wastewater, although the underlying mechanisms of degradation of organic pollutants are still unknown. In this study we employ two needle-type atmospheric pressure non-thermal plasma jets, i.e., indirect (ID-APPJ) and direct (D-APPJ) jets operating at Ar feed gas, for the treatment of methylene blue, methyl orange and congo red dyes, for two different times (i.e., 20 min and 30 min). Specifically, we study the decolorization/degradation of all three dyes using the above mentioned plasma sources, by means of UV-Vis spectroscopy, HPLC and a density meter. We also employ mass spectroscopy to verify whether only decolorization or also degradation takes place after treatment of the dyes by the NTP jets. Additionally, we analyze the interaction of OH radicals with all three dyes using reactive molecular dynamics simulations, based on the density functional-tight binding method. This investigation represents the first report on the degradation of these three different dyes by two types of NTP setups, analyzed by various methods, and based on both experimental and computational studies.

Due to the growing industrialization, the extensive use of chemicals led to an increase of the amount of unwanted pollutants in drinking water sources^1^,^2^,^3^,^4^,^5^. Every year Mediterranean olive-growing countries produce around 30 million m^3^ of highly toxic olive mill wastewater^6^. The water in these areas contains various impurities, such as heavy metals and organic pollutants - mainly dyes, pesticides, halogenated organic solvents, polychlorinated biphenyls (PCBs), and polycyclic aromatic hydrocarbons (PAHs)^7^. These pollutants are discharged from industries and wastewater treatment plants to natural water sources. Moreover, some pollutants, namely organic and synthetic dyes, are indispensably used not only within the dyeing and textile industries, but also in mechanical and electrical industries, such as in the production of solar cells, liquid crystal displays, and lasers^8^. These organic dyes are toxic to both flora and fauna when discharged into the environment, because such dyes absorb and reflect sunlight entering water sources, which results in the death of aquatic species, as well as bacteria that are used to degrade impurities in the water^8^,^9^,^10^. Hence, it is essential to degrade or decolorize these dyes before they are discharged into the water.

The large number of organic dye pollutants cannot be effectively mitigated by conventional wastewater degradation methods, because the molecular structures of these dyes are very stable and do not degrade easily. Therefore, to solve this problem, alternative methods, so-called advanced oxidation processes (AOPs), were developed over the previous years^11^. The most widely investigated AOPs are O_3_/UV, photo-fenton, photo-catalysis, and non-thermal plasma (NTP) methods^12^. Among these AOPs, the NTP method is studied most frequently because of the ability of NTP to generate various types of reactive oxygen and nitrogen species (RONS), such as OH, H, HO_2_, O_3_, NO, ONOO^−^, NO_2_^−^, NO_3_^−^, and H_2_O_2_, which have high oxidation potentials to react with stable organic dye molecules^3^,^13^,^14^,^15^,^16^,^17^,^18^.

There exist many different types of NTP devices, and among them DBD reactors are mainly used to degrade organic pollutants, such as AR88 acid^19^, acetone and toluene (by combination of TiO_2_ and DBD)^20^, azo dye Orange II^21^ and methyl orange^15^. Recently a DBD jet was used to degrade methylene blue^17^. In another work, Olszewski *et al*. studied the impact of pulse-modulated generation of atmospheric pressure plasma on the efficiency of methyl orange decolorization^22^. Moreover, in a recent review, Magureanu *et al*.^23^ focused on the degradation of pharmaceutical compounds in water by NTP, and showed the utility of NTP in wastewater purification. However, in only a few studies, more than one dye and more than one NTP source was used to study the dye degradation and/or decolorization. Furthermore, so far there are no studies demonstrating both the experimental and computational viewpoints on dye degradation.

Hence, in this study we apply two needle-type atmospheric pressure plasma jets (APPJs), i.e., an indirect (ID-APPJ) and a direct (D-APPJ) setup, both operating in Ar gas, for the treatment of methylene blue (MB), methyl orange (MO) and congo red (CR) dyes, for two different times (i.e., 20 min and 30 min). We investigate their decolorization/degradation process using UV-Vis spectroscopy, High-performance liquid chromatography (HPLC) as well as a density meter. We also analyze the final degradation products of these three dyes using Liquid Chromatography Tandem Mass Spectrometry (LC[QTOF]MSMS). Additionally, we study the interaction of OH radicals with the dyes, by means of reactive molecular dynamics (MD) simulations based on the density functional-tight binding (DFTB) method^24^.

## Results and Discussion

### Dyes degradation/decolorization and energy efficiency of the plasma devices

**Figure 1:**
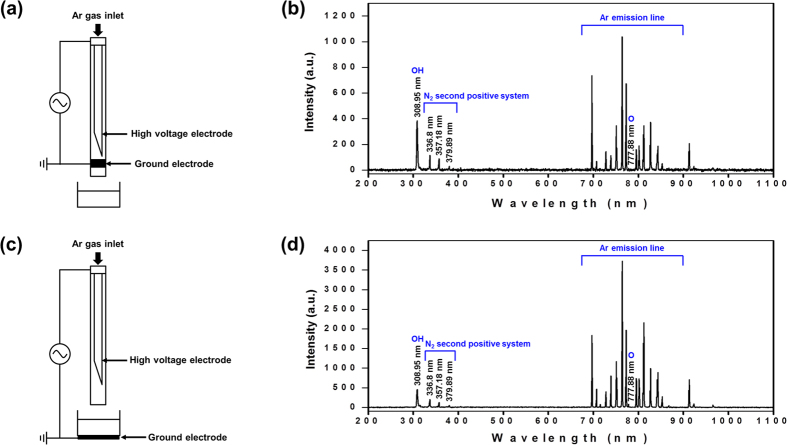
Schematic representation of the plasma sources used in this study, and the corresponding optical emission spectra, (**a**) ID-APPJ; (**b**) OES of ID-APPJ; (**c**) D-APPJ and (**d**) OES of D-APPJ.

**Figure 2:**
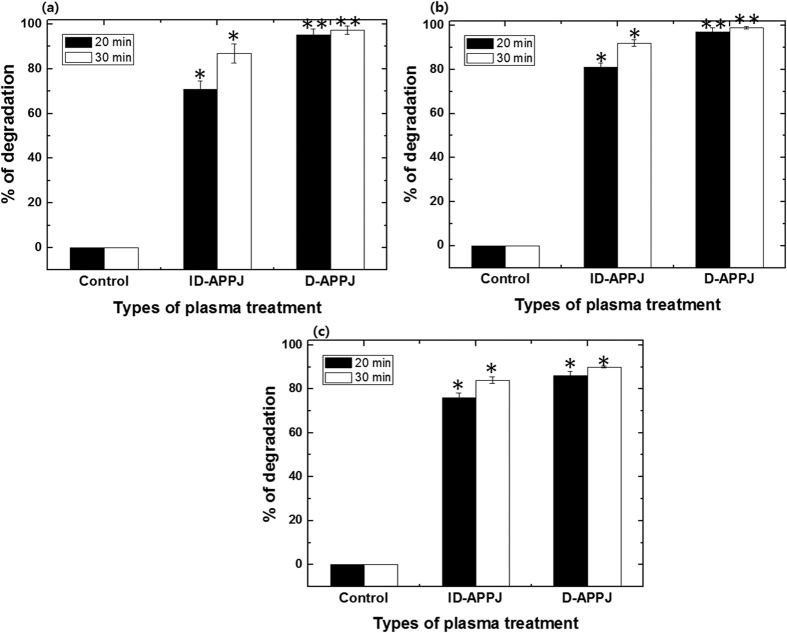
Degradation percentages obtained by UV-Vis spectroscopy for (**a**) MB, (**b**) MO and (**c**) CR, after treatment with ID-APPJ and D-APPJ for 20 min and 30 min.

The degradation of organic dyes, i.e., MB, MO and CR, by the ID-APPJ and D-APPJ setups is a complex process. The schematic illustrations of the both plasma sources (ID-APPJ and D-APPJ) and their corresponding optical emission spectra (OES), are displayed in Fig. 1. The voltage and current waveforms of both plasma devices are shown in Fig. S1. The plasma generates the primary reactive species in the gas phase and/or in the gas-liquid interface. They are transported to the liquid layer, which aids in the generation of secondary radicals due to their high reactive potential. These primary and secondary reactive species contribute to the breakdown of dye molecules into intermediate products or final stable products. In the present experiment we did not employ any forced stirring, so that the mixing in the system is limited to diffusion only. Figure 2 shows the degradation percentages of the MB, MO and CR dyes after treatment with the ID-APPJ and D-APPJ, for 20 min and 30 min, as obtained by UV-VIS absorption spectroscopy. In fact, we do not know yet for sure whether the process is really degradation or only decolorization, which is the visual effect. However, later we will elucidate that it is effectively degradation, and therefore, we already call it “degradation” for the sake of clarity.

The degradation of MB is 71% and 95% after the treatment with ID-APPJ and D-APPJ for 20 min, respectively, while it is 87% and 97% for 30 min of treatment, respectively (see Fig. 2a). In the case of MO and CR, the treatment with ID-APPJ and D-APPJ for 20 min resulted in a degradation of 81% and 97% (for MO) and 76% and 86% (for CR), respectively, whereas these percentages are 92% and 99% (for MO) and 84% and 90% (for CR), for 30 min of treatment time, respectively (see Fig. 2b,c). Similar trends were also observed by HPLC, and are shown in the Supporting Information (Figs S2–S5).

It is thus clear that the same plasma source cannot degrade all the dyes to the same extent. Additionally, the D-APPJ provides a stronger action in the degradation of all three dyes, compared to the ID-APPJ (see Fig. 2), which might be expected, because a direct source can provide more (short-living) reactive species to the solution. It should be mentioned that there is a difference in applied powers of both plasma sources, i.e., 0.2 W for the ID-APPJ and 0.4 W for the D-APPJ (see section 3.1), which might also explain the stronger action of the latter. However, because of the different powers used, it is also important to know which of the plasma devices is more energy efficient for the degradation of the dyes.

**Figure 3:**
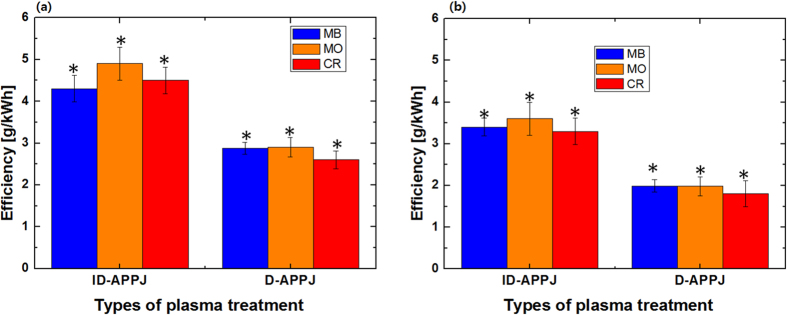
Energy efficiency of ID-APPJ and D-APPJ treatment of the three different dye solutions, for (**a**) 20 min and (**b**) 30 min.

Figure 3 illustrates the energy efficiency (g/kWh) of the ID-APPJ and D-APPJ setups, operated for 20 min and 30 min treatment of the dyes. The energy efficiency of the ID-APPJ setup for 20 min treatment of MB, MO and CR is 4.3, 4.9 and 4.5 g/kWh, respectively, while it is lower in the case of the D-APPJ setup, i.e., 2.8, 2.9 and 2.6 g/kWh, respectively (see Fig. 3a). For a 30 min treatment of MB, MO and CR, the energy efficiency is calculated to be 3.4, 3.6 and 3.3 g/kWh, respectively, for the ID-APPJ setup, whereas it is only 1.99, 1.98 and 1.8 g/kWh in the case of the D-APPJ setup (see Fig. 3b). Thus, for both treatment times, the energy efficiency is higher for the ID-APPJ setup than for the D-APPJ, despite the fact that the degradation was greater for the D-APPJ setup. This is explained because the power in the D-APPJ setup is twice as high as in the ID-APPJ setup, while the % degradation was not twice as high (cf. Fig. 2).

### Density of the dyes in the solution after plasma treatment

**Figure 4:**
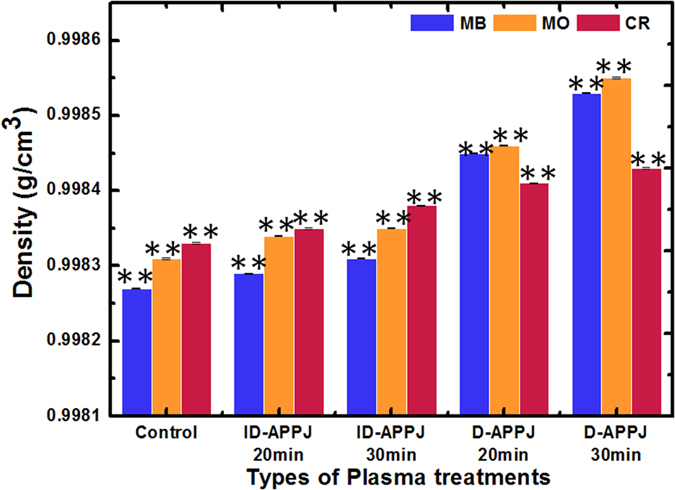
Density of the three different dye solutions, before and after plasma treatment with the ID-APPJ and D-APPJ setups for 20 and 30 minutes.

We measured the density of the solutions at 20 °C (see Fig. 4). The density of the control (i.e., untreated) MB is 0.99827 g/cm^3^, and after the treatment with ID-APPJ for 20 and 30 min, it slightly increases to 0.99829 and 0.99831 g/cm^3^, respectively. When the D-APPJ is applied for 20 and 30 min, the density of MB increases much more to 0.99845 and 0.99853 g/cm^3^, respectively. The same trend is seen for the density of MO. For CR, the D-APPJ also gives a increase in density, but the difference with the ID-APPJ effect is smaller (see Fig. 4). Thus, the density of the dyes increases after the treatment with plasma and the change in density is more pronounced for the D-APPJ compared to the ID-APPJ. Moreover, in both plasma sources the density of the dyes increases with increasing treatment time.

The increase in density can be due to the formation of some additional products or due to the intermolecular interactions between the degraded products. Thus, to analyze the degraded products, we performed LC[QTOF]MSMS after 30 min treatment with the D-APPJ setup, since the maximum degradation and the most pronounced increase in density of the dyes was observed at this condition.

### MS analysis for dyes degradation after plasma treatment

**Figure 5:**
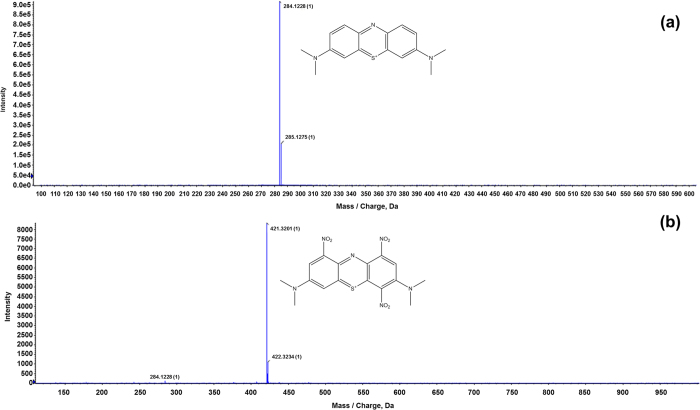
LC[QTOF]MSMS analysis for MB; (**a**) before and (**b**) after treatment with D-APPJ for 30 min.

**Figure 6:**
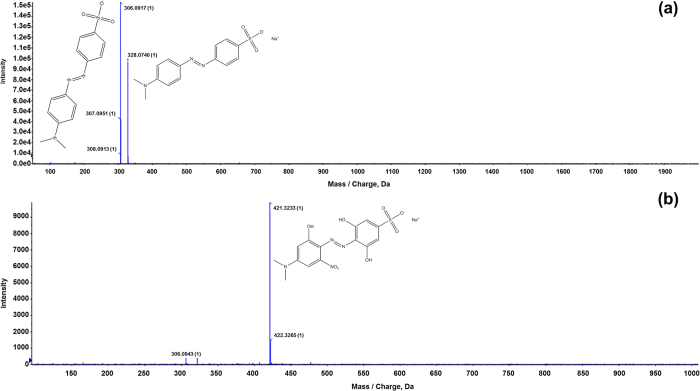
LC[QTOF]MSMS analysis for MO; (**a**) before and (**b**) after treatment with D-APPJ for 30 min.

**Figure 7:**
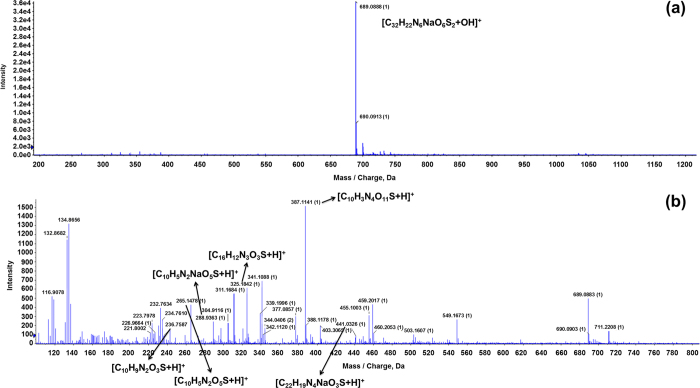
LC[QTOF]MSMS analysis for CR; (**a**) before and (**b**) after treatment with D-APPJ for 30 min.

We performed liquid chromatography (LC) separation for the MB, MO, and CR dye solutions, before and after 30 min treatment with the D-APPJ setup, and the subsequent TOF-MS spectra, taken from the same retention times before and after treatment, for the three different dyes are illustrated in Figs 5, 6 and 7. The results shown in Fig. 2 above indicate that the wastewater is significantly decolorized or degraded after 30 min irradiation with the D-APPJ setup. The current analysis helps us to understand whether the dyes are effectively degraded into small components or whether it is just a decolorization effect. For this purpose, we first checked the TOF-MS of the MB control sample from 50 to 2000 m/z (mass/charge) and observed the main peak at 284 m/z, that may belong to [C_16_H_18_N_3_S^+^ + H]^+^, as displayed in Fig. 5(a). However, after the treatment with D-APPJ, this peak has almost disappeared. Instead, a peak at 421 m/z, along with some small other peaks, appears after the plasma treatment. The peak at 421 m/z might correspond to [C_16_H_15_N_6_O_6_S^+^ + H]^+^, which might be generated from the reaction of reactive oxygen and nitrogen species (RONS) with the MB dye, resulting in nitration of MB (see Fig. 5b). The intensity of the newly generated peak at 421 m/z is around 10^4^, whereas the control peak at 284 m/z has an intensity of 10^6^ (i.e., 100 times higher, see Fig. 5). This newly generated product in the MB solution (after the D-APPJ treatment) is the reason for the slight increase in mass density of the MB solution from 0.99827 to 0.99853 g/cm^3^, as we see that the intensity of the new product is not high. Therefore, the change in density is only about 0.00026 g/cm^3^. From the TOF-MS data, we can conclude that the degradation of MB, as seen by absorption and HPLC spectroscopy (see Fig. 2 and Fig. S2), is really due to the degradation of the MB dye, and not only due to the decolorization.

We also performed TOF-MS analysis of the MO solution before and after treatment with the D-APPJ for 30 min (see Fig. 6). We again checked the peaks from 50 to 2000 m/z for the control MO solution and found the main peaks at 306 and 328 m/z (with intensities of 10^6^), which may belong to [C_14_H_14_N_3_O_3_^−^S + H]^+^ and [C_14_H_14_N_3_Na^+^O_3_^−^S + H]^+^. However, after the treatment with the D-APPJ setup, the peak at 328 m/z has disappeared and the peak at 306 m/z is reduced to the order of 10^3^. Moreover, new peaks appear at higher m/z, with intensities in the order of 10^4^, and the main peak appears at 421 m/z, which may be [C_14_H_13_N_4_Na^+^O_8_^−^S + H]^+^ generated due to hydroxylation and nitration of MO during the treatment with the D-APPJ setup. During the reaction of MO with RONS generated by plasma, we thus observe a degradation of the MO molecule into small fragments, but at the same time hydroxylation and nitration reactions take place with MO that form stable chemical compounds with a higher mass (or m/z) than MO itself. Similar to MB, the density of the MO solution also increases slightly after the plasma treatment, by 0.00024 g/cm^3^, which can be correlated to the small peaks appearing in the mass spectrum.

Finally, the TOF-MS of CR before and after D-APPJ treatment for 30 min is illustrated in Fig. 7. We again checked the peaks from 50 to 2000 m/z, and the main peak appears at 689 m/z (with intensity of 10^4^), which may belong to [C_32_H_22_N_6_Na^+^O_6_^−^S_2_ + OH]^+^. After the treatment with D-APPJ, the CR is also fragmented, and the peak at 689 m/z is reduced to the order of 10^3^. We observed many new peaks that appear with intensity around 10^3^ and 10^2^ (see Fig. 7). The new peaks at 441, 387, 325, 288, 265 and 236 m/z most likely correspond to the following fragments: [C_22_H_19_N4Na^+^O_3_^−^S + H]^+^, [C_10_H_3_N_4_Na^+^O_11_^−^S + H]^+^, [C_16_H_12_N_3_O_3_^−^S + H]^+^, [C_10_H_5_N_2_Na^+^O_5_^−^S + H]^+^, [C_10_H_5_N_2_O_5_^−^S + H]^+^ and [C_10_H_9_N_2_O_3_^−^S + H]^+^. This shows that plasma activated species can also degrade the CR dye. Moreover, there is not a significant change in the density data after the D-APPJ treatment, in comparison to the other 2 dyes (see Fig. 4 above). Indeed, before treatment, the density of the CR solution was 0.99833 g/cm^3^, while after treatment it is 0.99843 g/cm^3^. As mentioned above, some higher m/z products are formed from MB and MO after plasma treatment, but not so many products, and only fragments, are formed from CR. This explains why the increase in density of the CR solution after plasma treatment is lower than for the other dyes. The slight increase in density might be due to the intermolecular interactions between the degraded products. The fact that the main peak of CR decreases after D-APPJ treatment, corresponds to the absorption and HPLC studies mentioned above, and points towards degradation of the molecule, and not only decolorization. The hydroxylation and nitration reactions were also observed by our group and other groups during the plasma treatment of amino acids^25^,^26^,^27^.

In summary, all spectroscopy results (i.e., absorption, HPLC and TOF-MS) point out the degradation of the three different dyes after plasma treatment. In order to understand the mechanisms of the plasma degradation, we studied the generation of RONS in water after ID-APPJ and D-APPJ treatment for 30 min, as well as the interaction of OH radicals with the dyes using reactive MD simulations.

### Reactive species generation and change in physical properties of the solutions after plasma treatment

By means of OES, we try to understand the RONS production by the ID-APPJ and D-APPJ setups, using Ar feed gas. Figure 1b,d above show the typical spectrum of the plasmas generated from the ID-APPJ and D-APPJ setups, which interact with the ambient air with V_rms_ of 0.7 kV and 1.2 kV (see Fig. S1), respectively, and with an Ar flow rate of 3 L/m. The applied powers of these sources are 0.2 and 0.4 W, respectively. The emission lines, displayed in Fig. 1b,d, are identified according to the reported values^28^. We observe in these spectra the emission lines originating from OH radicals at 308.95 nm, the N_2_ second positive system peaks at 336.8 nm, 357.18 nm, and 379.89 nm, and atomic oxygen (O) at 777.88 nm. Additionally, we observe Ar emission lines at 696.44, 706.59, 727.35, 738.46, 750.32, 763.41, 772.46, 794.78, 826.51, 842.44, 852.14, 911.97, and 922.3 nm. The intensity of these Ar peaks is higher for the D-APPJ setup than for the ID-APPJ setup, which suggests that excited Ar* species are more generated in the D-APPJ setup than in the ID-APPJ setup. Hence, the density of radicals (generated from the reaction of Ar* species with H_2_O molecules) will also be higher in the case of the D-APPJ setup.

**Figure 8:**
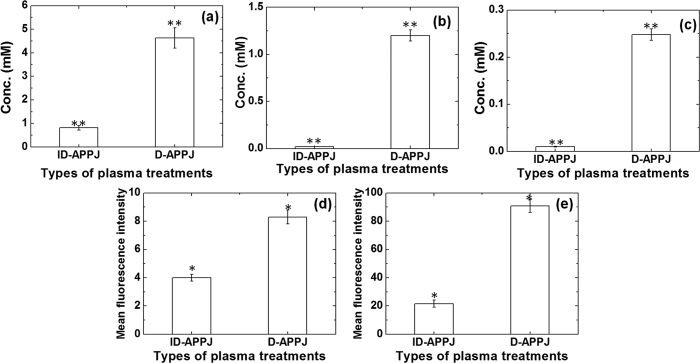
Analysis of various RONS in the solution, i.e., (**a**) H_2_O_2_, (**b**) NO_3_^−^, (**c**) NO_2_^−^, (**d**) OH and (**e**) NO, after treatment of DI water for 30 min with ID-APPJ and D-APPJ.

To understand the generation of RONS in DI water after treatment with the ID-APPJ and D-APPJ setups for 30 min treatment, we performed a chemical and electrochemical analysis. OH, NO, H_2_O_2_ and NO_2_^−^ were detected in DI water using chemical analysis, while NO_3_^−^ was detected with electrochemical analysis. It can be deduced from Fig. 8(a) that the concentration of H_2_O_2_ in the DI water after ID-APPJ and D-APPJ treatment is 0.8 mM and 4.6 mM, respectively. The NO_3_^−^ concentration is 0.02 mM and 1.2 mM (see Fig. 8(b)), and the NO_2_^−^ concentration is 0.01 mM and 0.24 mM (see Fig. 8(c)), after ID-APPJ and D-APPJ treatment, respectively. For the OH and NO radicals, we measured the mean fluorescence intensity; see Fig. 8(d,e). We can conclude from Fig. 8 that the concentration of all these RONS inside the water is higher after the D-APPJ treatment than after the ID-APPJ treatment. This is probably due to the higher power applied in the D-APPJ setup than in ID-APPJ setup.

**Figure 9:**
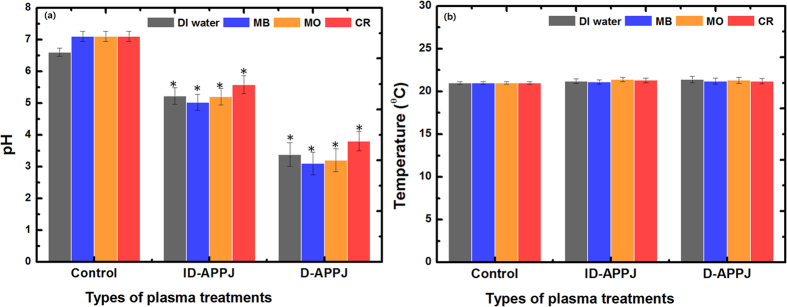
pH and temperature of DI water and the various dye solutions, before and after the treatment with ID-APPJ and D-APPJ for 30 min.

Further, we measured the pH and temperature before and after the plasma treatment of DI water, as well as the MB, MO and CR solutions, and the results are illustrated in Fig. 9. The pH values for all treated systems clearly drop, and the drop is most pronounced for the D-APPJ treatment. Moreover, the drop is somewhat lower for the CR solution and slightly higher for the MB solution. The smaller drop for CR might be due to the formation of NaOH in the CR solution after the plasma treatment, while the somewhat larger drop for MB is probably due to the formation of organic acids during the degradation process. On the other hand, the temperature did not change during the treatment in all systems, hence it seems that the temperature plays no role in the degradation of the dyes.

**Figure 10:**
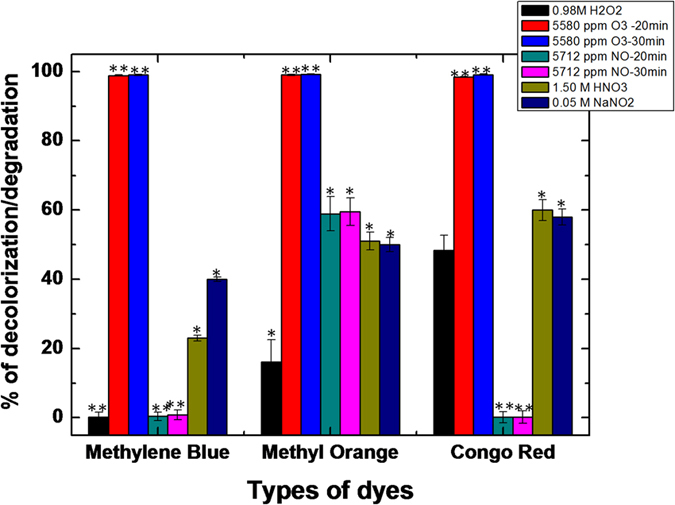
Decolorization/degradation percentages of the MB, MO and CR dye solutions after treatment with 0.98 M H_2_O_2_ (black), 5580 ppm O_3_ (red 20 min and blue 30 min treatment), 5712 ppm NO (cyan 20 min and magenta 30 min), 1.50 M HNO_3_ (yellow) and 0.05 M NaNO_2_ (purple).

We also treated all three dye solutions with 0.98 M H_2_O_2_, 1.50 M HNO_3_, 0.05 M NaNO_2_, 5712 ppm NO (20 min and 30 min) and 5580 ppm O_3_ (20 min and 30 min), to understand the impact of the various important RONS on the decolorization/degradation of the dyes; see Fig. 10. Note that the concentrations of these RONS are much higher than the RONS created in our plasma treatments. Indeed, as illustrated in Fig. 8 above, the H_2_O_2_ concentration in the DI water system was measured to be 4.6 mM at maximum, the maximum NO_3_^−^ concentration was 1.2 mM, and the maximum NO_2_^−^ concentration was 0.24 mM. Furthermore, the ozone concentration measured in the gas phase was 10 ppm at maximum, as mentioned in section 3.1. Finally, we also measured the NO fluorescence intensity in the three dye solutions after treatment with 5712 ppm, and its value was much higher than the fluorescence intensity generated through plasma (cf. Fig. 8(e)), using the same method (data not shown), which tells us that the NO concentration generated by the plasma treatment is indeed much lower than in the present case. For OH, we could not investigate the effect of a separate treatment, as we do not have a setup to separately generate OH radicals (see below).

Figure 10 shows that the treatment with 0.98 M H_2_O_2_ has no effect on the MB decolorization/degradation, while the decolorization/degradation is 20% and 50% for MO and CR, respectively. The treatment with 5712 ppm NO (20 min and 30 min) also shows no decolorization/degradation for MB and CR, but almost 60% for MO. However, the decolorization/degradation of MB, MO and CR is 23%, 51% and 60% after the treatment with 1.50 M HNO_3_, respectively. Moreover, the treatment with 0.05 M NaNO_2_ (standard solution for NO_2_^−^) yields 40%, 50% and 58% decolorization/degradation for MB, MO and CR, respectively. Finally, the treatment with 5580 ppm O_3_ (20 min and 30 min) has a very strong effect on the decolorization of all three dyes. In our plasma system the concentration of ozone is, however, only approximately 10 ppm in gas phase as mentioned above, which is very low compared to the concentration in this experiment. Hence, we expect that ozone will not have a large effect on the decolorization/degradation in our plasma treatments. Thus, from this figure, in combination with Fig. 2 above, we may conclude that the short lived radicals in the plasma treatment play an important role in degradation of the dyes. Among all the radicals, the OH radicals have the highest oxidation potential, i.e., 2.8 V, but we do not have a setup to generate only OH radicals, as mentioned above. Therefore, we performed reactive DFTB-MD simulations to study in detail the interaction of OH radicals with the dye molecules.

### Reactive DFTB-MD simulations

#### OH behavior in the surrounding water layer

When using the model systems surrounded by the water layer, the created OH radicals can also interact with the water layer itself. Our DFTB-MD simulations show that the OH radicals can indeed chemically react with the water molecules, exchanging a hydrogen atom and forming again the same species (i.e., a new OH radical and a water molecule), a process which is continuously repeated. The same behavior of the OH radicals in water was also observed in our previous work, by means of the classical reactive MD method, based on the ReaxFF potential^29^.

#### OH interaction with the dyes

**Figure 11:**
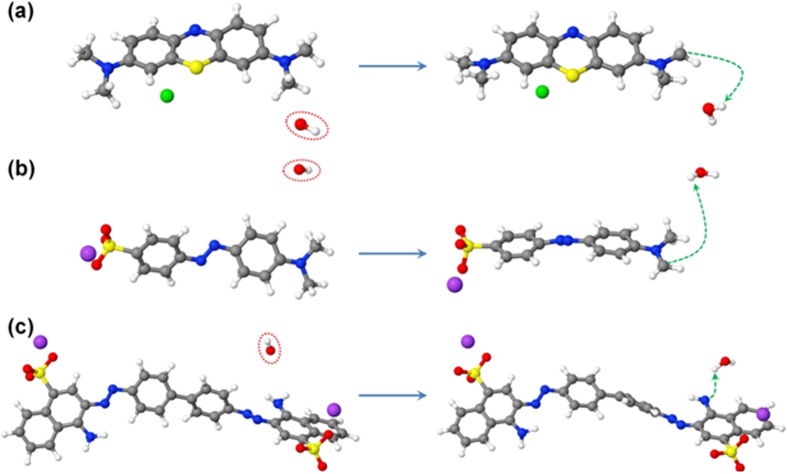
Snapshots from DFTB-MD simulations, showing the interaction of OH radicals with isolated MB (**a**), MO (**b**) and CR (**c**) molecules, leading to the formation of radical sites in the structures as a result of H-abstraction. The OH radicals approaching the structures are shown in red dashed circles and the H-abstraction reactions are presented by green dashed arrows. Note that the reactions shown in (**a**,**b**,**c**) are observed in 75, 36 and 49% of the simulated cases, respectively.

We observe several reaction mechanisms upon OH radical impacts. The most frequent reaction mechanism observed for each dye is presented in Fig. 11. In the case of MO and MB, the OH radical reacts with one of the methyl groups, abstracting a H atom and forming a water molecule as well as a CH_2_ radical site in the dye (see Fig. 11a,b), whereas in the case of CR, the OH radical abstracts a H atom from one of the amine groups, leaving behind an NH radical site (see Fig. 11c). These reaction mechanisms are observed in 36, 75 and 49% of the simulation cases for MB, MO and CR, respectively. These reaction mechanisms were also analyzed in the “real” water-stabilized structures, and the same reaction mechanisms were observed.

**Figure 12:**
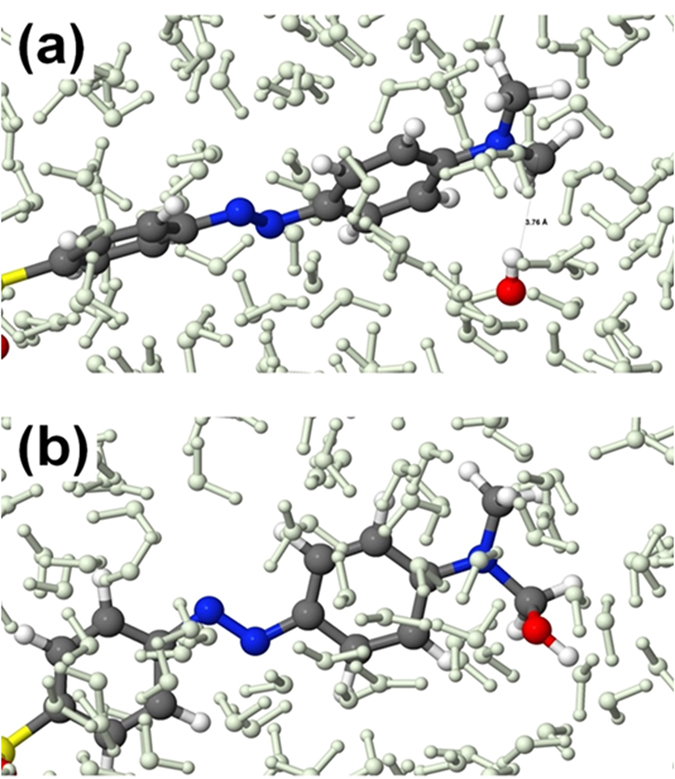
Snapshots from DFTB-MD simulations, showing the interaction of an OH radical with the radical site in MO, surrounded by water molecules. An OH radical is initially placed near the CH_2_ radical (i.e., at a distance of d_C-O_ = 3.76 Å, see (**a**)) and after travelling through the water layer (i.e., after ~185 fs) it finally reacts with this site and forms an alcohol group (**b**). The water molecules are shown in greyish green color, for the sake of clarity.

Subsequently, a new OH radical can react with these radical sites, forming an alcohol group (in the case of MO and MB) or a hydroxylamine group (in the case of CR). The formation of an alcohol group in MO surrounded by water is illustrated in Fig. 12. Indeed, it was found that after travelling through the water layer (possibly exchanging a H atom with a water molecule and forming a new OH), the OH radical finally reacts with the CH_2_ radical and forms an alcohol group, making the system more hydrophilic.

It should be mentioned that we do not really see degradation of the dyes or larger compound formation, as observed in the MS, but this is attributed to the limited time-scale of the simulations. In fact we expect further reactions if we would be able to run for much longer times. However, within the time-scale of the simulations, we already see reactions that alter the structure of the dyes, indicating indeed that OH radicals are able to destroy the molecules.

## Conclusion

We can conclude from our study that both plasma devices (i.e., the ID-APPJ and D-APPJ setups) have the potential to degrade all three dyes (MB, MO and CR), and the extent of decolorization/degradation is quite similar for the different dyes. The D-APPJ setup leads to more decolorization/degradation than the ID-APPJ setup, which can be explained by the fact that more (short-lived) reactive plasma species can be transferred to the dye solution (see below), but also by the higher power applied. When comparing the energy efficiency of both setups, the ID-APPJ gives better results, exactly because it operates at lower power, and still gives considerable degradation. A longer treatment time leads to somewhat more decolorization/degradation, but the effect is minor, and it also results in a lower energy efficiency. Therefore, the D-APPJ at longer treatment times might be the most appropriate, if as much as possible dye degradation is targeted. However, typically the energy efficiency is also equally important, and in that case, the ID-APPJ setup with 20 min treatment time is to be preferred, as it still yields a decolorization/degradation of 70–80% for the different dyes, at an energy efficiency of about 4–5 g/kWh.

Huang *et al*., obtained degradation for MB and MO of about 55% and 94% for 30 min DBD treatment, which is comparable but slightly lower than our results, but the energy efficiency was not determined by the authors^15^,^16^. In another work, an APPJ with Ar as feeding gas was used for the degradation of MB, and it was approximately 80% after 30 min treatment, while the efficiency was less than 0.4 g/kWh, thus clearly lower than in our experiments. Recently a DBD reactor was used to decolorize CR, but the decolorization was only 30% (100 ppm initial concentration) after 30 min treatment, which is clearly lower than in our case (using 200 ppm initial concentration), and the energy efficiency was not determined by the authors^30^. To our knowledge, there are no reports in literature yet where different plasma setups were compared for these dyes.

According to European drinking water regulations, the concentration of nitrate should be no more than 50 mg/l, and the maximum permissible limit of nitrite is 0.5 mg/l^31^. As illustrated in this work, the D-APPJ setup yields nitrate and nitrite concentrations in the solution of 74.4 mg/l (~1.2 mM) and 11.0 mg/l (~0.24 mM), while the ID-APPJ yields values of only 1.2 mg/l (~0.02 mM) and 0.46 mg/l (~0.01 mM), respectively, which are lower than the permissible limits. Therefore, we believe that the ID-APPJ setup might be more appropriate for water purification.

We also measured the density of the dye solutions after plasma treatment, and we observed some increase in density, being more pronounced for MB and MO, but the difference with the untreated solutions is in all cases very minor. Additionally, mass spectroscopy analysis showed that the plasma action has not only a decolorization effect, but also a degradation effect on the dyes, as new peaks appear in the MS.

To obtain a better insight in the underlying mechanisms and the role of various RONS in the plasma, we measured the concentration of various RONS in DI water after plasma treatment, and we could conclude that among the stable reactive species H_2_O_2_ is formed in the largest amounts, followed by NO_3_^−^ and NO_2_^−^. Furthermore, the D-APPJ setup yields clearly higher concentrations in the DI water than the ID-APPJ setup, which confirms our conclusion above, about the reason why the first setup yields more decolorization/degradation. Also the drop in pH is more pronounced in the D-APPJ setup. The temperature of the solutions, however, does not change for the different plasma setups and treatment times, indicating that it does not play a major role in this process.

A standard (non-plasma) treatment with several RONS showed that NO plays no role in the decolorization/degradation of MB and CR, while H_2_O_2_ has only effect for CR, and no or only a very minor effect for MB and MO, respectively. O_3_ seems to have a large effect on the decolorization/degradation of the three different dyes, but the concentration used in this treatment is much higher than in typical plasma treatments. We were not able to evaluate the separate decolorization/degradation potential of OH radicals, as we have no setup to generate them separately. Likewise, we could not quantify their concentration in the plasma-treated solutions, but from the fluorescence intensity, we know they are present in the solutions. Moreover, because we have obtained high concentrations of H_2_O_2_ in our plasma setups, and the OH radicals are the main components for the generation of H_2_O_2_, we may conclude that OH radicals are also generated in large amount in our plasma systems. Furthermore, among all the reactive species, the OH radicals have the highest oxidation potential. Hence, this suggests that the OH radicals are one of the main components in the plasma that lead to degradation of the dyes. Because we do not have a setup to generate only OH radicals, we could not draw firm conclusions, but we performed reactive DFTB-MD simulations to study in detail the interaction of OH radicals with the dye molecules, and we observed some reactions in all cases, leading to changes in the dye molecules. However, due to time scale limitations of the simulations, the product formation, as observed in the MS, could not be investigated.

In general we can conclude that the plasma treatment allows the degradation of the three different dyes, and not just one specific dye, and it has advantages over other techniques. Indeed, conventional biological treatments are ineffective for dye degradation due to the presence of aromatic rings in the dye molecules, and physical methods must transfer the organic pollutants from water to solid phase, which further requires post-treatment of the solid waste^32^. Additionally, the absence of byproducts, which are typically generated due to O_3_ and Cl_2_ treatment, also provides advantages for plasma treatment. Hence, this study provides new possibilities of plasma technology for water purification.

## Material and Methods

### Atmospheric pressure non-thermal plasma jet setups

The schematic illustrations of the ID-APPJ and D-APPJ setups, as well as their corresponding optical emission spectra (OES), are displayed in Fig. 1. The ID-APPJ source consists of a hollow inner needle electrode in a cylindrical glass tube supplied with Ar gas. The Ar gas flows along the hollow inner needle electrode at a flow rate of 3 l pm. The grounded electrode, which is made of copper tape with 2 mm width, is placed outside of the glass tube surface and located 1 mm away from the end tip of the inner electrode, as shown in Fig. 1a. Similarly, the D-APPJ consists of a hollow inner needle electrode in a cylindrical glass tube. The Ar gas, with 3 l pm flow rate, flows again through this hollow inner needle electrode covered with glass, but the grounded electrode (made of copper tape) is positioned below the sample, which is located 27 mm away from the end tip of the inner electrode (see Fig. 1c). The root mean square voltage (V_rms-_) is observed to be 0.7 kV and 1.2 kV, whereas the root mean square current (I_rms_) is 3 mA and 5 mA, for the ID-APPJ and D-APPJ setup, respectively. Both sources have a frequency of 16 kHz with a power of 0.2 W and 0.4 W, respectively. The power is calculated using the method reported in ref. 33. The voltage and current waveforms of both ID-APPJ and D-APPJ setups are shown in Fig. S1. The concentration of ozone, measured immediately after 30 min treatment with the ID-APPJ and D-APPJ setups, is found to be 2 and 10 ppm in the gas phase, respectively. The OES spectra of the ID-APPJ and D-APPJ emission are recorded by the HR4000CG-UV-NIR instrument (Ocean Optics, FL, USA) and optical fiber (QP400-2-SR) with a diameter of 400 mm, at humidity of 40%. UV spectra are measured over a wide wavelength range of 200–1100 nm in gas phase for both plasma devices. The signal is accumulated for 3 min, and the data are analyzed using the Origin 8.0 software package. The emission spectra are recorded as illustrated in Fig. 1b,d.

### Materials and analysis

A 1 g/L stock solution of MB, MO and CR is prepared by dissolving the required amount of analytical grade dye in Millipore water. The experimental solutions (200 mg/L) are obtained by diluting the stock solution in accurate proportions. Degradation of the dyes is monitored via a UV-Vis spectrophotometer S-3100, with a wavelength resolution, accuracy, and reproducibility of 0.95 nm, ±0.5 nm and ±0.02 nm, respectively. The absorption is measured at 660 nm, 460 nm, and 500 nm for MB, MO and CR, respectively, and the degradation percentage and energy efficiency (g/kWh) are calculated using a previously reported method^22^. The H_2_O_2_ concentration is measured using titanyl ions^34^,^35^ in the presence of sodium azide to control the H_2_O_2_ degradation by nitrites. The NO concentration is detected using 4-amino-5-methylamino-2′,7′-difluorofluorescein (DAF-FM)^36^, and the OH concentration is measured using terephthalic acid (20 mM) by means of the procedure described earlier in ref. 37. The NO_2_^−^ concentration is measured using Griess reagent supplied by Aldrich Chemical Co. (USA), whereas the NO_3_^−^ concentration is obtained using the Acorn Series ION 6 meter (pH/mV/°C Meter), nitrate electrode, from Oakton Instruments, USA. After exposure of both plasmas (generated from the ID- and D-APPJs) to deionized (DI) water and the three different solutions, the pH and temperature of the water and of the solutions are measured using a pH meter (Eutech Instruments, Singapore) and Infrared (IR) camera (Fluke Ti100 Series Thermal Imaging Cameras, UK). All measurements are performed in triplicate. The density of the dyes in the solution is measured using an Anton-Paar DSA 5000 with an accuracy in temperature ±0.01 K, whereas the uncertainty in the density is ±0.00005 g cm^−3^. Prior to the measurements, the instrument is calibrated with DI water and dry air as standards at 293.15 K^38^,^39^,^40^.

The filtrates are measured by a high performance liquid chromatography method using HPLC-UV (Agilent 1200, USA) containing a ZORBAX SB-C18 column (2.1 mm 150 mm, 5 mm). 30/70 (v/v) of acetonitrile/10% acetic acid is used as the mobile phase for MB, MO and CR in HPLC-UV. The analysis is performed at 25 °C with 0.8 mL/min flow rate of an injected 100 mL volume of sample. The final degradation products are analyzed using LC[QTOF]MSMS. The LCMSMS consists of a TripleTOF 5600 (quadrupole-Time of flight) tandem mass spectrometer (ABSciex). The TOF mass range is 40,000 m/z with a maximum resolution power of MS 25,000@m/z 195, MS/MS 35,000@m/z 965, and a mass accuracy of <0.05 ppm.

To understand the effect of various RONS, generated by the plasma, on the decolorization/degradation of the dyes, we also treat the dye solutions with ozone and nitric oxide. Ozone (O_3_) is generated using an ozone machine (Model: LAB-II, Company: OZONE TECH). Applying this technique we generate 5580 ppm of ozone, the concentration of which is measured using the detection tubes obtained from Gastec (Product No. 18M and 18L, Gastec, Japan). These tubes contain a reagent which changes its color after coming into contact with the ozone. Note that these tubes have an accuracy of about ±10% due to the presence of other interfering species. Nitric oxide (NO) is generated using a microwave plasma system. This system consists of a magnetron, waveguide component (WR-340 for 2.45 GHz) and a microwave plasma torch apparatus, as described in our earlier work^41^, which can generate 5712 ppm of NO.

### Simulation details

We use the so-called DFTB3 method, which is the extended and improved version of self-consistent charge DFTB^42^. For the description of the interatomic interactions in our DFTB-MD simulations, we employ a recently developed parameter set, called ‘3ob-3-1’^43^,^44^. In the experiments the dye molecules are surrounded with a water layer. However, due to the high computational cost of the DFTB method, the simulation of the entire system, including the water layer, requires a prohibitively long calculation time. Moreover, to study in detail all possible bond breaking and formation processes upon OH radical impact, and to gain some (limited) statistics, we need to perform a large number of DFTB-MD simulations. Therefore, we consider the model systems in vacuum, and perform 100 DFTB-MD simulations for each dye molecule; hence, 300 DFTB-MD runs are conducted in total. Subsequently, we also analyze the most often occurring reactions, as obtained from the vacuum simulations, by performing simulations for the “real” water-stabilized structures, i.e., the systems in a water environment.

**Figure 13:**
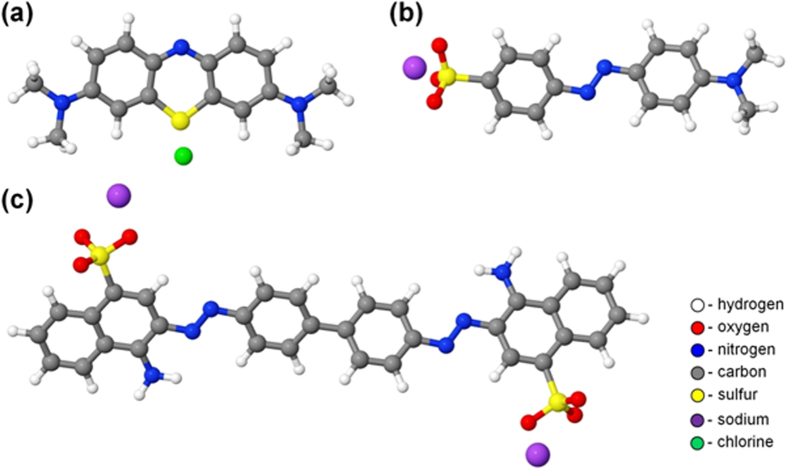
Schematic pictures of the dye molecules, i.e., MB (**a**), MO (**b**) and CR (**c**), used in the simulations. The color legend also applies to the other Figs. below, related to the simulations.

Figure 13 displays the model systems (in vacuum), which are used to study the reaction mechanisms and to gain some limited statistics. The MO and MB molecules (containing 36 and 39 atoms, respectively) are each placed in a box with size of 25 Å × 25 Å × 25 Å, whereas CR (containing 70 atoms) is positioned in a larger simulation box, i.e., with size of 30 Å × 30 Å × 30 Å. These box sizes are large enough to randomly create a single OH radical around the structures. The geometry of the systems is optimized using the conjugate gradient algorithm. The systems are then equilibrated for 25 ps in an NVT ensemble (i.e., a system with constant number of particles N, volume V and temperature T), at 300 K, employing the Berendsen thermostat^45^ with a coupling constant of 100 fs. We use a time step of 0.5 fs in all simulations, i.e., during the thermalization, as well as during the particle impact simulations. Periodic boundary conditions are imposed in all three directions. Subsequently, a single OH radical is randomly created around the structure with a minimum distance of 5 Å from the system. This is done to avoid initial interactions between the OH radical and the system due to long distance interactions (i.e., Coulomb and van der Waals interactions). The impact simulations (i.e., 100 DFTB-MD runs for each structure) are performed for a total simulation time of 30 ps (i.e., 6 × 10^4^ iterations) per simulation, which is long enough to realize bond breaking and formation in the structures.

After the calculations with these structures in vacuum are completed, we then use the water-stabilized structures (i.e., the structures in a water environment) for the analysis of the most frequent reactions. In these cases we place the OH radical closer (at ~2 Å) to the position where it should react (according to the vacuum simulations), to avoid excessively long calculation times in the DFTB method. The systems surrounded by water are also prepared in the same way as the aforementioned method. However, they are placed in smaller boxes (i.e., 20 Å × 20 Å × 20 Å for MO and MB, and 25 Å × 25 Å × 25 Å for CR, respectively), as the OH radical is placed closer to the structures. The structures are also thermalized using the NVT ensemble, but for a shorter simulation time, i.e., 5 ps, due to the high computational cost of the DFTB method. Our calculations show that this time is sufficient for equilibration of the systems. Subsequently, MD simulations are performed, but now for only 5 ps (i.e., 10^4^ iterations), due to the severe computational cost of DFTB. Since the OH radicals are positioned close enough to their reacting positions, this time is sufficient for the reactions to occur.

### Statistical analysis

All values are represented by the mean ± S.D of the indicated number of replicates. Statistical analyses of the data were performed using student’s t-test to establish the significance between data points, while the significant differences were based on *P < 0.05 and **P < 0.01.

## Additional Information

**How to cite this article**: Attri, P. *et al*. Mechanism and comparison of needle-type non-thermal direct and indirect atmospheric pressure plasma jets on the degradation of dyes. *Sci. Rep.* **6**, 34419; doi: 10.1038/srep34419 (2016).

## Supplementary Material

Supplementary Information
