# Supplementary material for: Mechanism and comparison of needle-type non-thermal direct and indirect atmospheric pressure plasma jets on the degradation of dyes
Source: Sci Rep. 2016 Oct 6;6:34419. doi: 10.1038/srep34419 (PMC5052639; doi:10.1038/srep34419)
Supplement: Supplementary Information [file srep34419-s1.pdf]

Mechanism and comparison of the needle-type non-thermal direct and indirect atmospheric pressure plasma jets on the degradation of dyes

Pankaj Attri[a,b*#], Maksudbek Yusupov[c#], Ji Hoon Park[a#], Lakshmi Prasanna Lingamdinne[d], Janardhan Reddy Koduru[e], Masaharu Shiratani[b], Eun Ha Choi[a*], and Annemie Bogaerts[c*]

[a]Plasma Bioscience Research Center / Department of Electrical and Biological Physics, Kwangwoon University, Seoul 139-701, Korea.

[b]Graduate School of Information Science and Electrical Engineering, Kyushu University

Fukuoka 819-0395, Japan

[c]Research Group PLASMANT, Department of Chemistry, University of Antwerp, Universiteitsplein 1, B-2610 Antwerp, Belgium

[d]Department of Environmental Engineering, Kwangwoon University, Seoul, 139-701, Republic of Korea

[e]Graduate School of Environmental Studies, Kwangwoon University, Seoul, 139-701, Republic of Korea

Supporting file Figure captions

Fig. S1: Waveforms of current and voltage for both ID-APPJ and D-APPJ

Fig. S2: Degradation percentages calculated by HPLC for (a) MB; (b) MO and (c) CR, after treatment with ID-APPJ and D-APPJ for 20 min and 30 min.

Fig. S3: HPLC graphs for MB; (a) control, (b) and (c) after ID-APPJ treatment for 20 min and 30 min, respectively, and (d) and (e) after D-APPJ treatment for 20 min and 30 min, respectively.

Fig. S4: HPLC graphs for MO; (a) control, (b) and (c) after ID-APPJ treatment for 20 min and 30 min, respectively, and (d) and (e) after D-APPJ treatment for 20 min and 30 min, respectively.

Fig. S5: HPLC graphs for CR; (a) control, (b) and (c) after ID-APPJ treatment for 20 min and 30 min, respectively, and (d) and (e) after D-APPJ treatment for 20 min and 30 min, respectively.

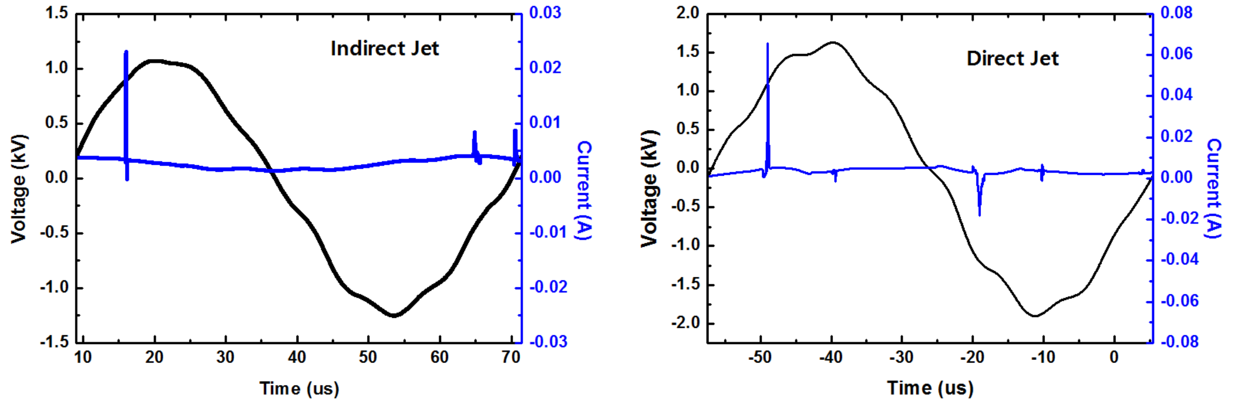

Fig. S1

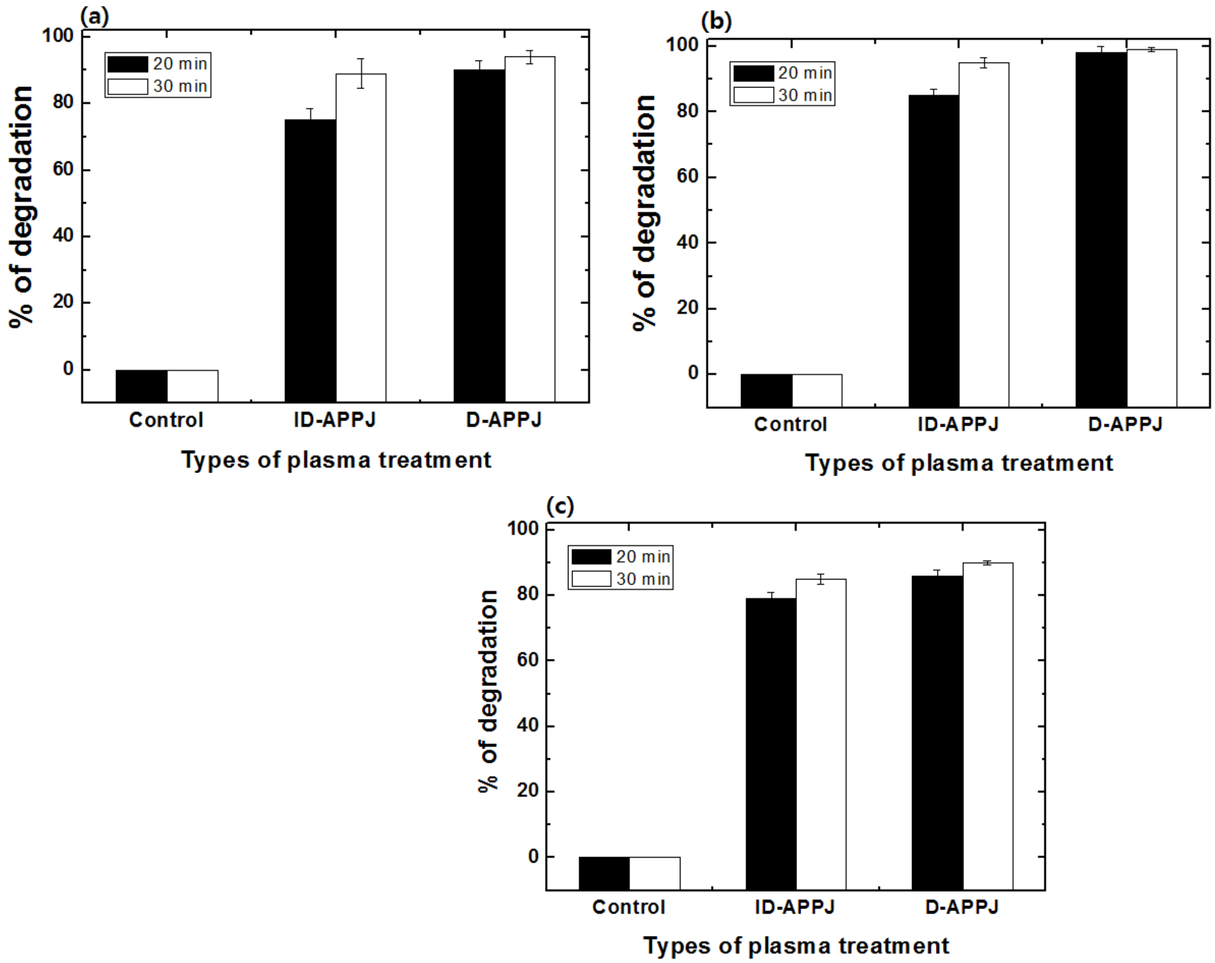

(a)
(b)
(c)
20 min
30 min
% of degradation
100
80
60
40
20
0
Control
ID-APPJ
D-APPJ
Types of plasma treatment

Fig. S2

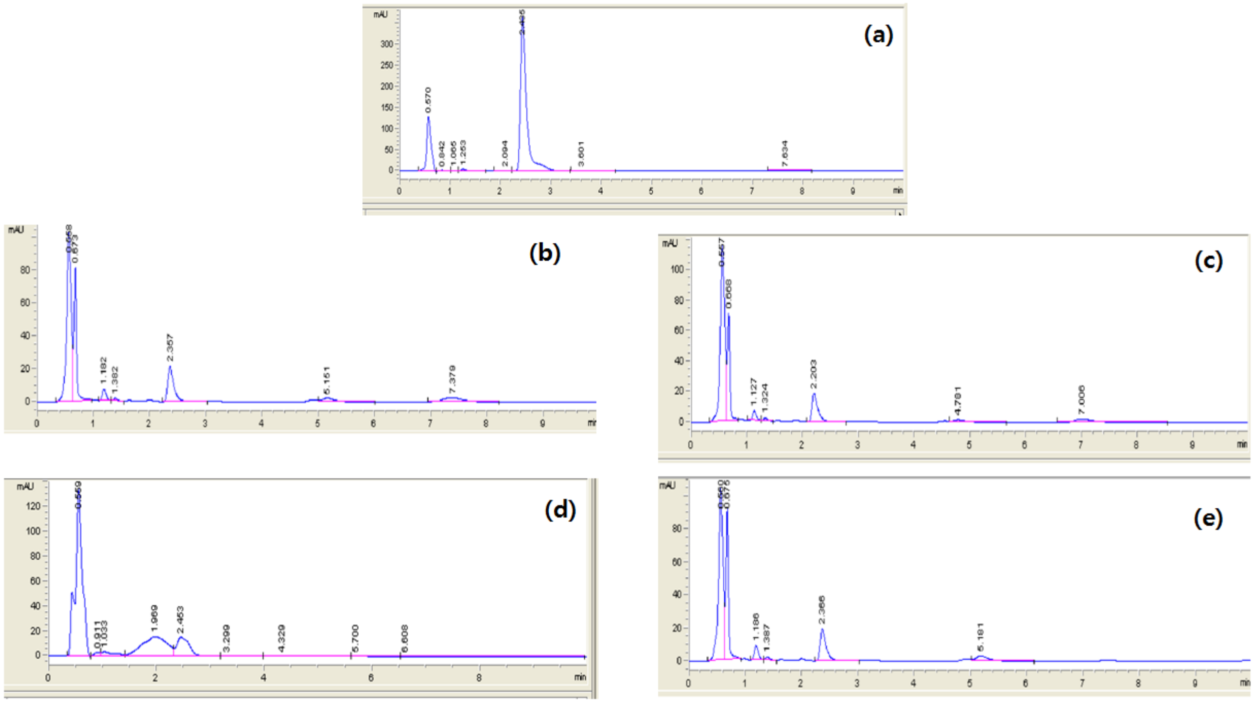

Fig. S3

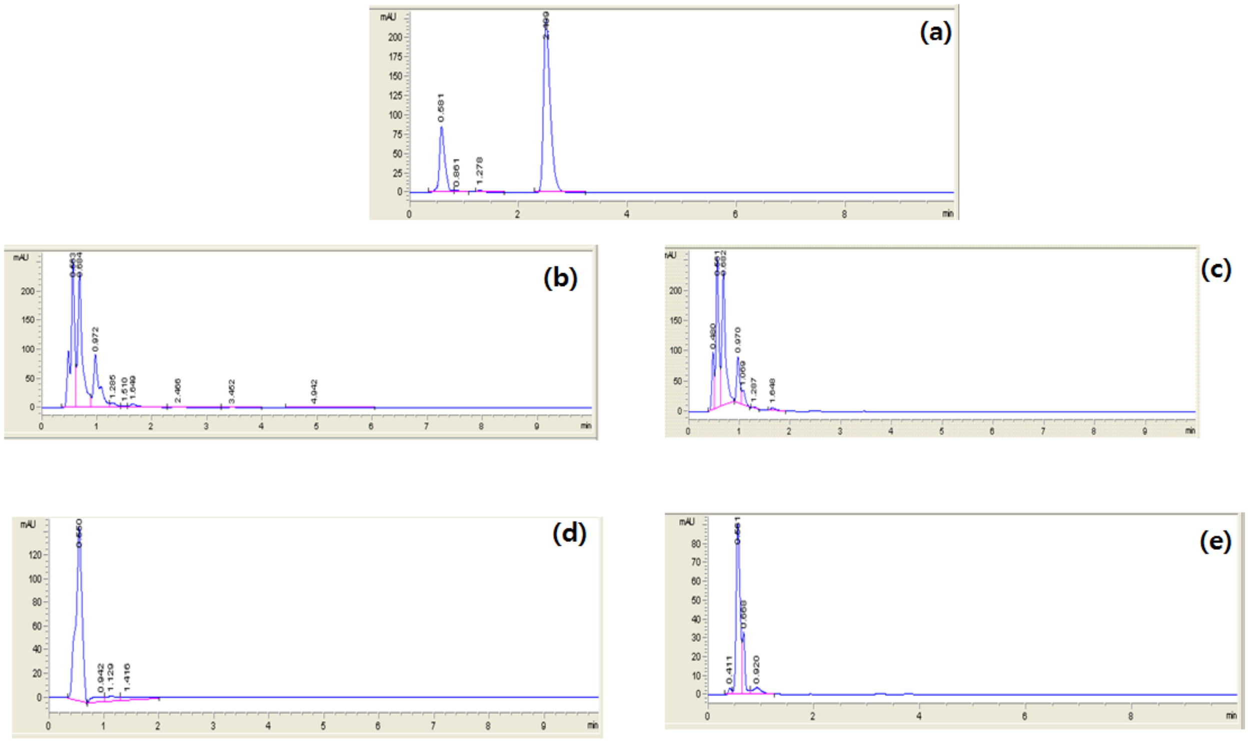

Fig. S4

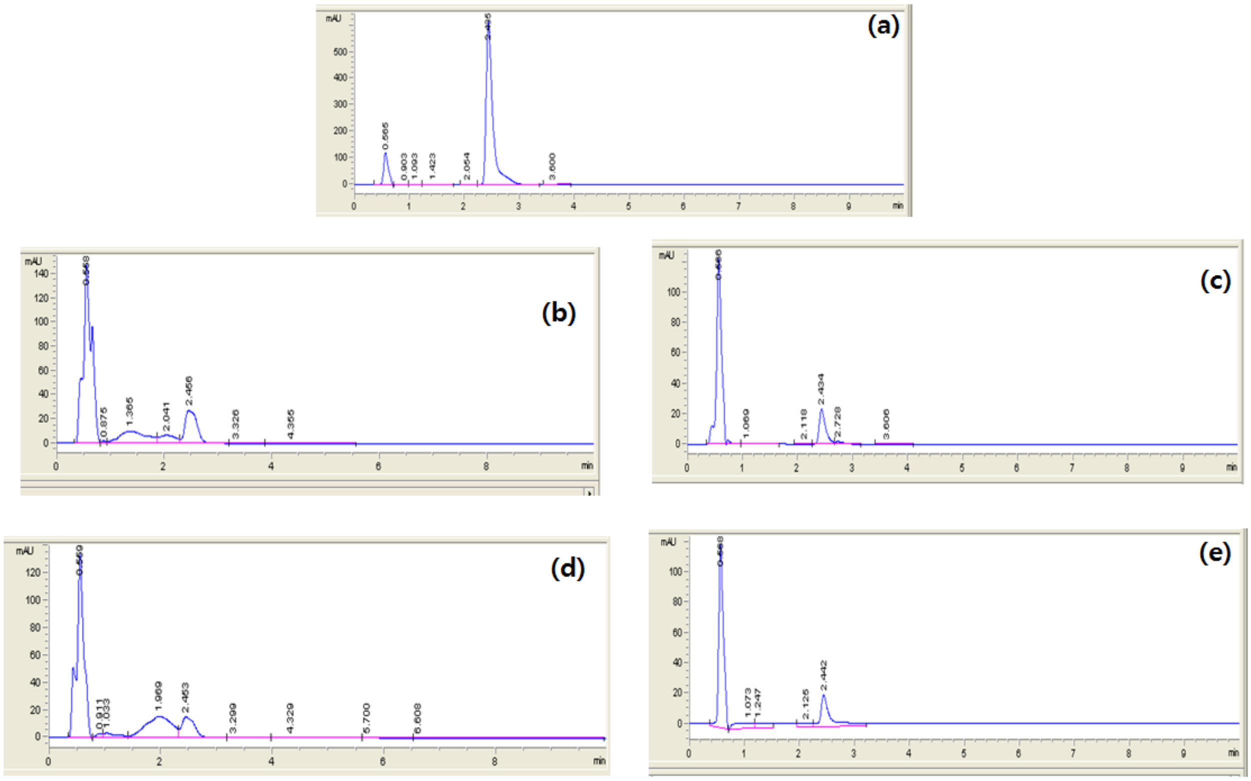

Fig. S5
